# Azorean Black Tea (*Camellia sinensis*) Antidermatophytic and Fungicidal Properties

**DOI:** 10.3390/molecules28237775

**Published:** 2023-11-25

**Authors:** Chantal Fernandes, José Sousa-Baptista, Ana Filipa Lenha-Silva, Daniela Calheiros, Edmilson Correia, Artur Figueirinha, Lígia Salgueiro, Teresa Gonçalves

**Affiliations:** 1 CNC-UC—Center for Neuroscience and Cell Biology of Coimbra, University of Coimbra, 3004-504 Coimbra, Portugal; uc41498@uc.pt (C.F.); josesousabaptista@gmail.com (J.S.-B.); ana.flmsilva@gmail.com (A.F.L.-S.); danielamcalheiros@gmail.com (D.C.); edcorreia17@gmail.com (E.C.); 2 CIBB—Centre for Innovative Biomedicine and Biotechnology, University of Coimbra, 3004-504 Coimbra, Portugal; 3 FMUC—Faculty of Medicine, University of Coimbra, Rua Larga, 3004-504 Coimbra, Portugal; 4 Faculty of Pharmacy, Health Sciences Campus, University of Coimbra, Azinhaga de S. Comba, 3000-548 Coimbra, Portugal; amfigueirinha@ff.uc.pt (A.F.); ligia@ff.uc.pt (L.S.); 5 Associated Laboratory for Green Chemistry (LAQV) of the Network of Chemistry and Technology (REQUIMTE), University of Porto, 4099-002 Porto, Portugal; 6 Chemical Process Engineering and Forest Products Research Centre, Department of Chemical Engineering, Faculty of Sciences and Technology, University of Coimbra, 3030-790 Coimbra, Portugal

**Keywords:** *Camellia sinensis*, Azorean Black Tea, antidermatophytic, antifungal, dermatophytes, cell wall, chitin, glucan, ergosterol

## Abstract

The treatment of dermatophytoses, the most common human fungal infections, requires new alternatives. The aim of this study was to determine the antidermatophytic activity of the aqueous Azorean Black Tea extract (ABT), together with an approach to the mechanisms of action. The phytochemical analysis of ABT extract was performed by HPLC. The dermatophytes susceptibility was assessed using a broth microdilution assay; potential synergies with terbinafine and griseofulvin were evaluated by the checkerboard assay. The mechanism of action was appraised by the quantification of the fungal cell wall chitin and β-1,3-glucan, and by membrane ergosterol. The presence of ultrastructural modifications was studied by Transmission Electron Microscopy (TEM). The ABT extract contained organic and phenolic acids, flavonoids, theaflavins and alkaloids. It showed an antidermatophytic effect, with MIC values of 250 µg/mL for *Trichophyton mentagrophytes*, 125 µg/mL for *Trichophyton rubrum* and 500 µg/mL for *Microsporum canis*; at these concentrations, the extract was fungicidal. An additive effect of ABT in association to terbinafine on these three dermatophytes was observed. The ABT extract caused a significant reduction in β-1,3-glucan content, indicating the synthesis of this cell wall component as a possible target. The present study identifies the antidermatophytic activity of the ABT and highlights its potential to improve the effectiveness of conventional topical treatment currently used for the management of skin or mucosal fungal infections.

## 1. Introduction

Dermatophytes are a group of filamentous fungi capable of colonizing keratinized structures [1]. They may cause a polymorphic spectrum of superficial infections, medically termed as dermatophytosis [2]. Although not life-threatening, dermatophytosis represent a noteworthy unsolved global public health problem. In fact, it is amongst the most common contagious skin diseases (around 20% to 25% of the world population) and is associated with significant morbidity and economic implications [3,4,5,6,7,8]. The treatment of dermatophytosis remains challenging because most of the current chemotherapeutic options comprise considerable limitations. The existing drugs approved for its treatment have limited capacity for eradicating the dermatophytes from the infected skin tissues [9,10,11]. Moreover, the rising resistance to the current antifungal armamentarium may jeopardize the treatment [12,13,14]. These phenomena seem to be related to the frequent relapse and reinfection, which poses an increasing difficulty on its clinical management [15]. Therefore, there is still a medical need for more effective antifungal strategies for treating dermatophytosis, and natural products have been a fruitful source for the discovery of new drugs [16]. 

Tea, obtained from *Camellia sinensis,* (L.) Kuntze is the second most consumed beverage worldwide after water, and accumulating evidence has linked tea consumption to various health benefits [17]. Different important pharmacological properties of tea have been reported, such as antimicrobial, antioxidant, and anti-inflammatory activities [18]. The plant *C. sinensis* is native to Southeast China and has gradually expanded into many tropical and subtropical countries. Since the last decade of the 19th century, it is also sustainably and eco-friendly cultivated in the volcanic São Miguel Island of the Azores Archipelago (Portugal) [19]. The antifungal activity of *C. sinensis* tea on yeast and filamentous fungi has been described [20,21,22,23]. *C. sinensis* extracts have also shown antifungal activity in vitro and in vivo in rats against *Candida* spp. [24]. The tea antifungal action has been attributed to polyphenols [25]. Green tea and black tea are obtained from the *C. sinensis* plant by different fermentation processes [26]. During the fermentation process, most catechins of the tea leaves are oxidized to theaflavins and thearubigins [27]. Therefore, black tea also contains a large number of widely recognized compounds with pharmacological value and therapeutic applications [28]. Several studies have demonstrated the antifungal activity of black tea against phytopathogenic fungi [29] and namely against different species of *Candida* [30,31]. Moreover, the anticandidal activity of *C. sinensis* fermented tea was described to be higher than the activity of non-fermented tea [32]. It was also shown that the black tea polyphenols, catechins and theaflavins have antifungal activity [30]. However, little is known about its antidermatophytic activity. Only few reports show the in vitro activity of black tea against dermatophytes and some of its constituents have been identified as having an inhibitory effect against *Trichophyton* spp., namely catechins and theaflavin [33,34].

The present study was designed to characterize the phytochemical composition of Azorean Black Tea (ABT) and to investigate the ABT extract activity on selected dermatophytes, representing the most common species agents of dermatophytosis. To unravel its mechanisms of action and the synergy between ABT and terbinafine and griseofulvin, the most used antidermatophytic drugs used in human health, were also evaluated.

## 2. Results

### 2.1. ABT Extract Preparation and Yield of the Extraction

The determination of dry weight of the ABT extract enabled us to determine that the concentration of extract obtained was between 4000 and 4500 µg/mL. To uniformize all the extract concentrations, we diluted all the extracts to 4000 µg/mL prior to use. The yield of the extraction was η = 18.2 ± 2.5% (mean ± SD).

### 2.2. Phytochemical Analysis of ABT Extract

The phytochemical analysis of the ABT extract was performed using high-performance liquid chromatography coupled with photodiode array and electrospray mass spectrometry detectors (HPLC–PDA–ESI–MSn). The chromatogram obtained from the MS detector is represented in Figure S1.

All data used for the identification of compounds are included in Table 1. The identification was based on spectroscopic information generated from the two detectors used. The absorption maxima extracted from the ultraviolet/visible (UV/vis) spectra (λmax) were used to identify the phytochemical class of compounds. This information was complemented with mass spectrometry data (ESI–MSn), namely the pseudomolecular ion allows for the inference of the molecular mass of the compounds, and the fragmentation pattern (MS2 and MS3) was related to the chemical structure. Several classes of compounds were identified in ABT extract, namely organic acids (isocitric acid); phenolic acids, including hydroxycinnamic and gallic acid derivatives; flavonoids, including monomeric and oligomeric flavan 3-ols; flavones and flavonols; phenolic acids; theaflavins; and alkaloids (Table 1).

#### 2.2.1. Organic Acids

Based on the fragmentation pattern proposed by Masik and co-workers (2017), Peak 1 (pseudomolecular ion at *m*/*z* 191) was identified as isocitric acid [35]. The mass spectra of peak 1 exhibited the diagnostic fragments at *m*/*z* 173, 155 and 111.

#### 2.2.2. Gallic Acid Derivatives

Peaks 2, 3 and 7 exhibited a UV maxima near 270 nm, and pseudomolecular ions at 343, 169 and 633, respectively, were identified as gallic acid derivatives based on their fragmentation behavior previously described in the literature [36]. Peak 2 produced MS2 fragment ions at *m*/*z* 191 and 169, corresponding to the deprotonated ions of quinic acid and gallic acid, which is consistent with a galloylquinic acid structure. Peak 3 corresponded to gallic acid (*m*/*z* 169), originating a typical loss of CO_2_ and producing an ion at *m*/*z* 125 [M−H-44]− as the characteristic fragment. Peak 7 (*m*/*z* 633) exhibited two main fragments at MS2 spectra: the fragment at *m*/*z* 463 from the loss of a gallic acid unit (170 amu) followed by the loss of a hexose unit (162 amu) originating the fragment at *m*/*z* 301. This fragmentation pattern is consistent with the presence of a galloyl-HHDP-glucose that was previously reported in black tea [37].

#### 2.2.3. Flavan 3-Ols

The sample chromatogram exhibited six peaks with a spectra behavior that could be related to flavan 3-ols. Peaks **5** (*m*/*z* 761), 6 (*m*/*z* 305), 9 (*m*/*z* 457), 10 (*m*/*z* 457), 16 (*m*/*z* 441) and 17 (*m*/*z* 441) were identified on the basis of their absorbance spectrum (UV max near 279 nm) and MS fragmentation pattern. Peak 5 was assigned as a (epi)gallocatechin dimmer esterified with a gallic acid unit. This structure is consistent with the mass spectra behavior, showing a pseudomolecular ion at *m*/*z* 761 and a fragmentation pattern consisting with fragment *m*/*z* 609 from the loss of a gallic acid unit, *m*/*z* 591 for the loss of water. The proposed structure for peak 5 was (epi)gallocatechin-(epi)gallocatechin gallate, which was previously found in black tea extracts [38]. In peak 6, the pseudomolecular ion at *m*/*z* 305 originated the fragment ion at *m*/*z* 261 from the loss of CO_2_, and also fragments 221 (loss of C_4_H_4_O_2_), 219 (loss of C_4_H_6_O_2_) and 179 (loss of C_6_H_6_O_3_). This behavior was previously reported for (Epi)gallocatechin also identified in black tea samples [36]. Peaks 9 and 10 had the same pseudomolecular ion at *m*/*z* 457 and the same fragmentation pattern, exhibiting a main fragment at *m*/*z* 169 corresponding to a gallic acid unit. Other fragments include 305 (gallocatechin unit). This is a typical behavior of a gallocatechin gallate that could exist in the form of steroisomers, which, however, could not be distinguished by mass spectrometry. Despite this, due to its behavior in reversed-phase chromatography, the compound corresponding to peak 9 can be identified as epigallocatechin gallate, while peak 10 will be gallocatechin galate. These compounds were previously identified in black tea by other authors [36]. Peaks 16 and 17 were tentatively identified as (Epi)catechin gallate isomers. The two peaks showed the same deprotonated ion at *m*/*z* 441 and produced fragment ions at *m*/*z* 289 and 169 corresponding to the deprotonated ion of catechin (or epicatechin) and gallic acid, respectively. Due to its chromatographic behavior the first eluted compound is the epi isomer (peak 16), while the second (peak 17) could be identified as catechin gallate [36].

#### 2.2.4. Flavones

Based on the UV and mass spectra, peaks 12, 13, 14 and 15 were identified as flavone derivatives, namely apigenin glycosides. Peak 12 exhibited a pseudomolecular ion at 593 and a fragmentation pattern consistent with C-glycosilation flavones. The presence of fragments at *m*/*z* 503 (loss of 90 amu) and 473 (loss of 120 amu) indicates the presence of a C-hexosyl unit). The same losses were observed in the MS3 spectra (fragments at *m*/*z* 320 and 353) as a result of another C-hexosyl. Therefore, this compound was tentatively identified as 4″-*O*-Glucosylvitexin since it shares the same spectral characteristics, and it was also previously identified in black tea [36]. Peaks 13, 14 and 15 share the same spectral characteristics, with a pseudomolecular ion at *m*/*z* 563 and fragments at *m*/*z* 503 (loss of 60 amu) and 473 (loss of 90 amu) at MS2 from a C-pentosyl unit and in MS3 with fragments at *m*/*z* 383 (loss of −90 amu) and 353 (loss of −120 amu) from a C-hexosyl unit. The higher abundance of the pentosyl unit suggests its location in C6 and the hexosyl unito in C8. Therefore, the proposed structure for the isomers is 6-pentosyl–8 hexosyl–apigenin.

#### 2.2.5. Hydroxycinammate Quinic Esters

Peak 8 exhibited a pseudomolecular ion at *m*/*z* 337 and was identified as *p*-coumaroylquinic acid based on UV spectra and fragmentation patterns reported for the caffeoylquinic acid isomers. The fragmentation of this peak, yielding a majority MS^2^ product ion at *m*/*z* 173, was coherent with 4-*O*-*p*-coumaroylquinic acid. The presence of hydroxicinamic acids in black tea is common; however, this was the only identified in our sample [36].

#### 2.2.6. Flavonols

Twelve flavonols were identified in the sample according to their UV spectra and MS fragmentation patterns. Five peaks were quercetin derivatives, originating the quercetin aglycone at *m*/*z* 301 in negative mode, while the other eight peaks were kaempferol derivatives (*m*/*z* 285). Peaks 18, 19 and 20 exhibited the same UV spectra and pseudomecular ion (*m*/*z* 771), originating a fragment at *m*/*z* 609 from the loss of a hexosil unit (162 amu) followed by the loss of 308 amu, corresponding to a hexosil-deoxyhexosil dissacaride, attached to the quercetin aglicone (*m*/*z* 301). Kebelek (2016) previously found quercetin-3-*O*-galactosyl-rhamnosyl-glucoside in black tea, and this kind of structure could be also present in our samples [36]. Peaks 21 and 22 were also quercetin derivatives. In this case, they were diglicosides since presented a pseudomolecular ion at 609 and fragment at 301 from the loss of a hexosil-deoxyhexosil unit (308 amu). Peaks 23 and 24 had a pseudomolecular ion at 755 and were tentatively identified as kaempferol-3-*O*-glucosyl-rhamnosyl-glucoside based on their UV spectra and mass fragmentation pattern with fragments at *m*/*z* 593 and 205 from the loss of a hexosyl and a hexosil-deoxyhexosil unit, respectively. Peaks 25 and 26 had the same pseudomolecular ion at *m*/*z* 593 and a main fragment at *m*/*z* 285. This behavior is characteristic of kaempferol (MW 286) bounded to a hexosil-deoxyhexosil unit (308 amu). Peaks 27 and 28 were identified as O-hexosil kaempferol (MW 448). This is consistent with the pseudomolecular ion at *m*/*z* 477 and the fragment at 285 from the loss of a hexosyl unit (162 amu). Finally, peak 29 has a pseudomolecular ion at *m*/*z* 1033 and fragment at *m*/*z* 887 and 741. In the MS3 spectra, there was a fragment at *m*/*z* 301. This fragmentation pattern was previously described in black tea for quercetin-3-(2G-*p-coum-trans*-3G)-2G-arabinosyl-3R-rhamnosyl rutinoside [36].

#### 2.2.7. Theaflavins

Peaks 30 and 31 were identified as Theaflavin gallate, on the basis of the MS data and a comparison with the literature [39]. The diagnostic fragments of theaflavins were produced primarily through Retro-Diels–Alder (RDA) fission (C_7_H_6_O_3_, 138 Da) with the loss of H_2_O and galloyl moiety (Gall, 152 Da). Based on their elution order in reversed-phase chromatography peaks 30 and 31 were identified as theaflavin 3-gallate and theaflavin 3’-gallate.

#### 2.2.8. Purine Alkaloids

Peaks 4 and 11 exhibited the same UV maxima (273 nm) but different pseudomolecular ions in the mass spectra in positive ion mode: *m*/*z* 181 and 195 corresponding to theobromine (MW 180) and caffeine (MW 194). This spectral behavior was previously observed by other authors in studies related to black tea [36].

Several polyphenolic compounds, such as the catechins galloyl-(epi)gallocatechin-(epi)gallocatechin, (epi)gallocatechin, (epi)gallocatechin gallate and the theaflavin gallate, as well as methylxanthine caffein, have been identified in the ABT extract, and since catechins and caffein are described for their antifungal activity [30,40], the antidermatophytic activity of the ABT extract was evaluated.

### 2.3. Antidermatophytic Activity of ABT Aqueous Extract

Antifungal susceptibility testing was performed to determine the minimal inhibitory concentration (MIC) of the ABT, following the EUCAST E.DEF 9.3.1 standards for filamentous fungi (https://www.aspergillus.org.uk/wp-content/uploads/2016/03/EUCAST_E_Def_9_3_Mould_testing_definitive_0.pdf, accessed on 9 November 2022). The MIC values of the ABT were 250 µg/mL for *Trichophyton mentagrophytes*, 125 µg/mL for *Trichophyton rubrum* and 500 µg/mL for *Microsporum canis* (Table 2). In order to use ABT extract preparations at the same concentration (4000 µg/mL) for all the assays, the antifungal activity of a fresh and frozen ABT extract was tested. The fresh and the frozen ABT extract presented the same MIC values (results not shown). The MIC determined for terbinafine and griseofulvin (Table 2) were in accordance with the literature [41,42]. 

To understand whether the ABT extract was fungicidal or fungistatic against these dermatophytes, the minimal fungicidal concentration (MFC) was determined. An antifungal agent is considered fungicidal if the ratio of MFC/MIC does not exceed a value of 4; otherwise, it is considered fungistatic [43,44]. The MFC determined for each fungal species was equal to the MIC (Table 1), indicating that the ratio of MFC/MIC is 1, meaning that the ABT extract can be considered fungicidal. 

### 2.4. Synergy between ABT and Antifungals Clinically Used

The synergistic activity of ABT with the clinically used antifungal agents, namely, terbinafine and griseofulvin, was evaluated by the checkerboard assay method. The fractional inhibitory concentrations Index (FICI) obtained for each combination and fungi tested is shown in Table 2. The combination of terbinafine and ABT had an additive effect on the three dermatophyte species tested. In other words, the effect of the combination of terbinafine and ABT is equal to that of the sum of the effects of the individual components. Yet, the combination of griseofulvin and ABT had an indifferent effect on the three dermatophytes tested, which means that the result of this combination was equal to the action of the most active component.

### 2.5. Modulation of Fungal Cell Wall ß-1,3-glucan and Chitin, and Fungal Cytoplasmic Membrane Ergosterol in Response to ABT

In order to unravel the possible mechanism of action underlying the ABT antidermatophytic effect, the β-1,3-glucan, chitin and ergosterol contents of *T. mentagrophytes, T. rubrum* and *M. canis* were quantified. 

**Figure 1:**
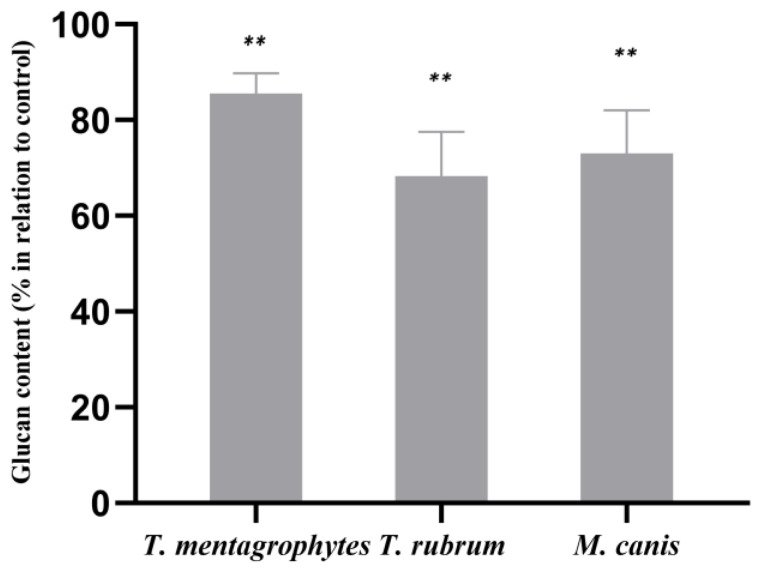
Effect of ABT on the cell wall β-1,3-glucan contents of *T. mentagrophytes, T. rubrum* and *M. canis*. The results, expressed in percentage in relation to control, are the means ± SEMs from three independent experiments performed in triplicates. **, *p* < 0.05. Data were normalized to control values.

The growth of the dermatophytes in the presence of ABT extract led to a statistically significant decrease in the β-1,3-glucan content. The decrease in β-1,3-glucan content was 14.5% in *T. mentagrophytes,* 31.8% in *T. rubrum* and 27.0% in *M. canis* when compared with control (Figure 1).

**Figure 2:**
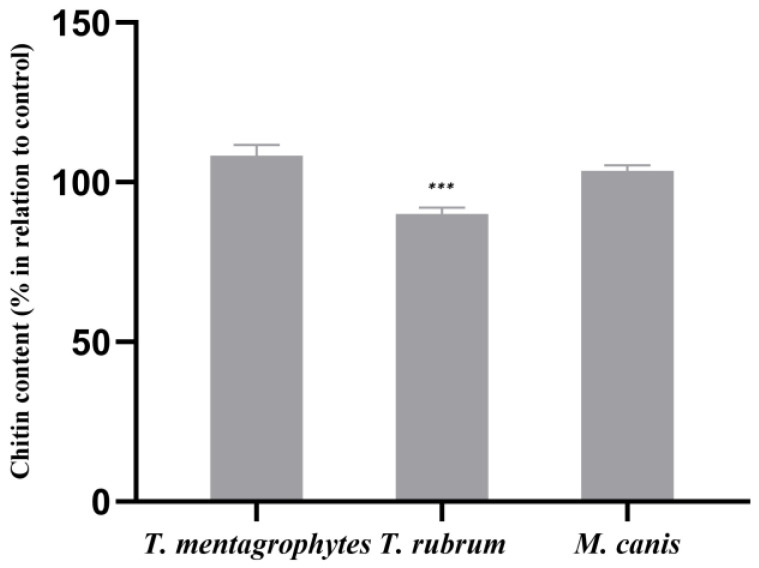
Effect of ABT on the cell wall chitin contents of *T. mentagrophytes, T. rubrum* and *M. canis*. The results, expressed in percentage in relation to control, are the means ± SEMs from three independent experiments performed in triplicates. ***, *p* < 0.001. Data were normalized to control values.

The quantification of the chitin contents in *T. rubrum* grown with the ABT extract revealed a statistically significant decrease of 10.0% of this cell wall component compared to the control (Figure 2). In *T. mentagrophytes* and *M. canis*, the ABT extract led to a slight increase in chitin contents with no statistical significance. 

**Figure 3:**
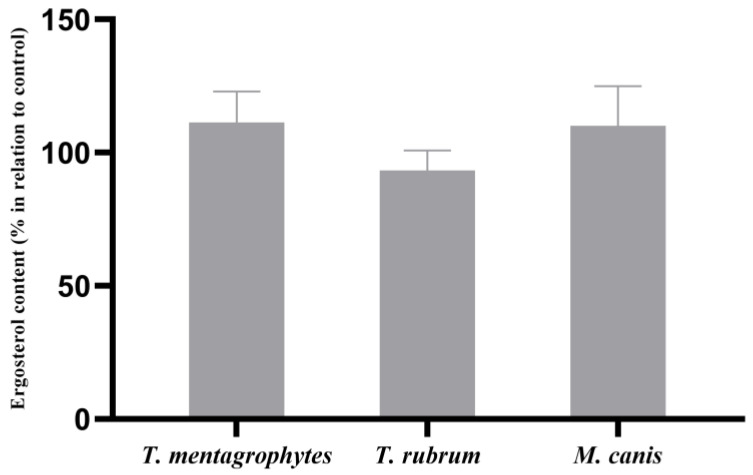
Effect of ABT on the cell wall ergosterol contents of *T. mentagrophytes, T. rubrum* and *M. canis*. The results, expressed in percentage in relation to control, are the means ± SEMs from three independent experiments performed in triplicates. Data were normalized to control values.

Otherwise, the ABT extract did not induce significant modifications in the ergosterol content of the fungal cell membranes of any of the tested fungi when compared to cells growing in the absence of ABT (Figure 3). 

### 2.6. Characterization of Morphological and Ultrastructural Changes

Transmission electron microscopy (TEM, JEOL, Tokyo, Japan) was used to appraise the ultrastructural changes in the hyphae of fungi grown in ABT-supplemented media. *T. mentagrophytes* and *M. canis* were selected because these presented the highest and the lowest susceptibility to ABT, respectively. 

**Figure 4:**
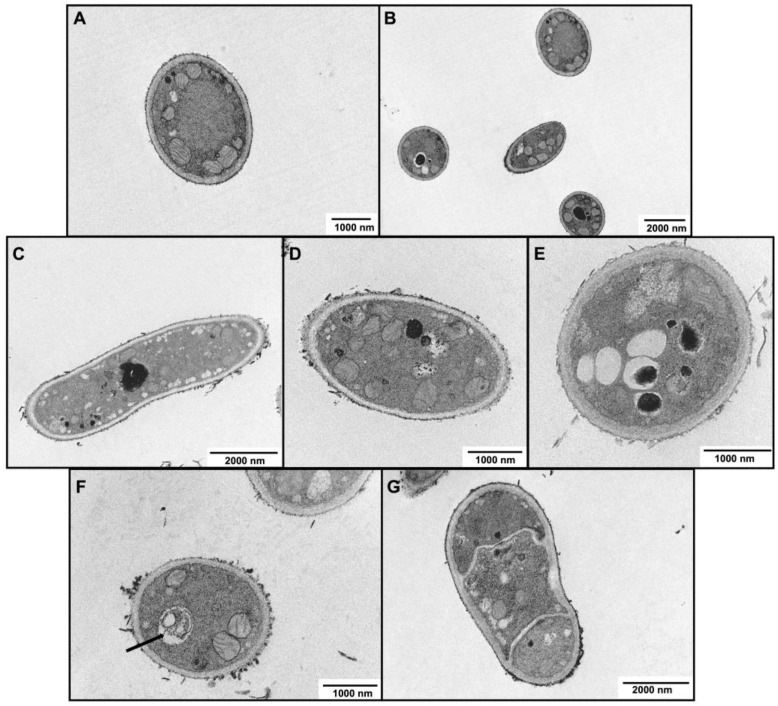
Transmission electron microscopic observations of the hyphae morphology of *T. rubrum* following exposure to Azorean Black Tea. (**A**,**B**) Growth control and (**C**–**G**) with 125 μg/mL ABT. Black arrow indicates an autophagy-like vacuole.

Untreated cells of *T. rubrum* appeared to be morphologically intact and presented numerous mitochondria with well-defined cristae (Figure 4A,B). The cells were delimited by a well-defined cell wall (Figure 4A,B). When *T. rubrum* was grown in ABT-supplemented media, the inner part of the cell wall was less electron-dense and at the surface some debris was accumulated (Figure 4C–G). ABT-treated cells presented numerous small vacuoles (Figure 4C,E) that in some cases contained electron-dense material (Figure 4E). In Figure 4F, autophagy-like vacuoles can be seen (black arrow). Moreover, the mitochondria cristae were not well defined as in the control (Figure 4D,F). 

**Figure 5:**
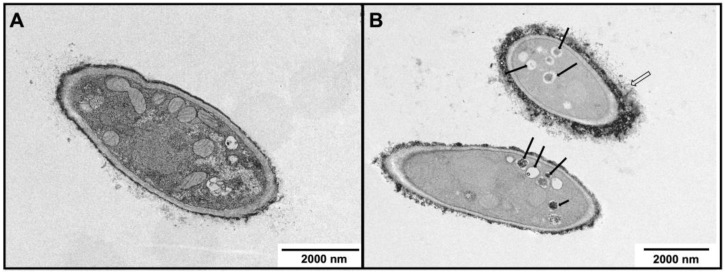
Transmission electron microscopic (TEM) observations of the morphology of *M. canis* hyphae following exposure to ABT. (**A**) Control, in the absence of ABT and (**B**) with 500 μg/mL ABT. Open arrow indicates electrodense material at the cell wall surface and black arrows indicate vacuoles with debris.

*M. canis* grown in control conditions exhibited smooth surfaces, a regular cell wall composed of an electron-dense outer layer, intact cytoplasmic membranes, and structured organelles and with a regular membrane (Figure 5A). In contrast, in the presence of ABT, several ultrastructural modifications were observed (Figure 5B), including deep modifications at cytoplasm and its components, especially mitochondria, that were less abundant. An increase in the vacuolization was also noticeable, and some of them containing debris (black arrows) (Figure 5B). The inner part of the cell wall layer was less electron transparent than in the control and the cell wall surface was coated by an electron-dense material, most probably not corresponding to the fungal cell wall but to tea pigments (Figure 5B).

## 3. Discussion

*C. sinensis* teas are known for their health benefits and by their antimicrobial activities [45]. Four kinds of teas can be obtained from the leaves of this plant according to the processing: white tea; green tea (unfermented); yellow tea, and red tea (semi-fermented); and black tea and dark tea (pu-erh), which are fully fermented [46,47]. These teas have different chemical compositions, characteristics of flavor, aroma and color [47]. 

In the present work, we studied the antidermatophytic effect of black tea because despite several previous studies having demonstrated the antifungal action of this tea, only scarce data are available about its bioactivity against dermatophytes. The black tea used was obtained from the unique place in Europe where tea is produced, São Miguel Island in the Azores Archipelago (Portugal), an island with an oceanic climate and mineral-rich volcanic soils appropriate for the cultivation of *C. sinensis*. The processing conditions to obtain the final product, black tea from the Azores, were previously optimized and characterized, [48,49]. Our approach was to perform the phytochemical analysis of the infusion obtained from a commercial Azorean Black Tea, produced by one of the brands of Azorean Tea, Porto Formoso. The extract was prepared under the conditions used for consumption. Since a previous study compared cold and hot extractions of black tea against *T. mentagrophytes* and concluded that the hot extraction had higher activity than cold extraction [50], and that the use of hot water in the preparation of tea infusion is more popular [51], aqueous ABT extract was prepared by hot extraction from this commercially available tea in approximately the same conditions as it is prepared for tea consumption. The concentration of these aqueous extracts was about 4000 µg/mL, as determined by the dry weight. The phytochemical analysis of this extract revealed the presence of some catechin derivatives and theaflavins, namely theaflavin 3-gallate and theaflavin 3′-gallate. Theaflavins are known to be present in black teas, since it results from the fermentation process that leads to the oxidation of the catechins to theaflavins [27], which present antifungal activity [30]. Caffein was also identified in the ABT extract, which was expected because black tea is known to contain this compound [52], and also presents antifungal activity [40]. Theaflavins, catechins and caffein were also reported in Azorean Black Tea from other producers (the Gorreana Tea Factory), and their proportions vary depending on the plant part used and on the fermentation time and temperature [49]. Also, flavan 3-ols and their esterified derivatives like (epi)gallocatechin gallate and (epi)catechin gallate for example, were detected in higher amounts than expected. According to the literature, most of these catechins are usually converted into theaflavins during the black tea fermentation process [27]. The flavonoid component of the ABT extract comprise several kaempferol derivatives and quercetin derivatives, which is in accordance with the literature that report that kaempferol and quercetin represent the main fraction of black tea flavonoids [53]. Interestingly, the flavonoids kaempferol and quercetin have antifungal activity against *Candida* spp. [54].

We studied the in vitro antidermatophytic effect of ABT extract and its potential mechanism of action on *T. mentagrophytes, T. rubrum* and *M. canis*, which are considered the three most common species found worldwide as agents of dermatophytosis [55]. The in vitro antidermatophytic effect of ABT extract was determined using a microdilution broth, following EUCAST standards, to determine the MIC of the ABT on the three dermatophyte species. *T. rubrum* was the most susceptible to ABT extract followed by *T. mentagrophytes*; *M. canis* was the dermatophyte with the lowest susceptibility. Previously, a study comparing the antifungal activity of *C. sinensis* black tea and green tea on *T. mentagrophytes* reported a MIC_90_ of 3125 µg/mL for green tea. For black tea, the authors could not quantify the MIC because it was above the highest concentration tested [50]. However, we cannot compare both works because Cheruiyot and collaborators determined the concentration of the tea in relation to the weight of the initial tea (10 mg in 100 mL of water) and not from the concentration of the final extract, after filtration and the removal of the herbs, as was performed in the present work. On the other hand, the duration of the extraction was not mentioned. Due to the lack of data about the antidermatophytic activity of black tea, it is only possible to compare the results obtained by us with the antidermatophytic effect of compounds found in the ABT used in our study. In fact, epigallocatechin 3-*O*-gallate (EGCG) presents an MIC of 8 µg/mL against *M. canis* and 4 µg/mL against *T. mentagrophytes* and *T. rubrum*, which is in the MIC range of clinically used antifungals [34], indicating that this compound has a remarkable antifungal activity. The lower MIC obtained using EGCG is due to the purity of the compounds in relation to the ABT extract that contains this compound among others. Our ABT extract enriched in catechin derivatives, including EGCG, was revealed to be fungicidal to *T. mentagrophytes*, *T. rubrum* and *M. canis.* So the ABT extract is not only fungistatic, but also effectively causes fungal death. This fungicidal activity was also demonstrated before against *Trichophyton* spp. with Lipton^®^ tea (*Camellia sinensis* black tea) and by its component, theaflavin-3,3′-digallate (TF3) [33], although the methodologies used by these authors cannot also be compared to the present work with ABT extract. The antidermatophytic activity of caffeine has also been demonstrated before against *T. mentagrophytes* (our unpublished results).

Antifungal combinations in the treatment of infections by dermatophytes have been shown as a promising strategy to accelerate the clinical healing of a superficial infection [56]. In fact, the present study revealed that there is an additive effect with the combination of the ABT extract and terbinafine but not with griseofulvin. Terbinafine is an allylamine that targets the fungal cell membrane by inhibiting one of the enzymes that leads to the formation of ergosterol, which leads to the disruption of the membrane by the accumulation of compounds that should be converted to ergosterol, while griseofulvin acts in the fungal cell, impairing mitosis [57]. This additive interaction observed between terbinafine and ABT may be related to the additive effects of compounds acting on membrane ergosterol and on cell wall components, respectively. The association between drugs and natural products may minimize the adverse effects of reducing the dose required to obtain the same effect. Previous studies showed antifungal synergy between conventional antifungals and compounds found in black tea. In *C. albicans*, amphotericin increases the permeability of the cell membrane, which leads to an increase in catechins entry, with increased antifungal effect [58]. Also, in *Candida* spp., it has been shown that EGCG synergistically enhanced the antifungal potential of azole drugs [59]. However, this is the first report of black tea, a complex preparation containing several bioactive compounds, improving the effect of terbinafine against dermatophytes.

As an attempt to unravel the therapeutic target of ABT extract, we quantified the modulation of the ergosterol, β-1,3-glucan and chitin contents from these dermatophytes grown in the presence and absence of ABT. ABT did not modify the levels of ergosterol, the main sterol in the cell membrane of fungi, essential to ensure the activity of membrane-bound enzymes, allowing for cell membrane permeability, fluidity and stress resistance [60,61]. However, a non-significant decrease in *T. mentagrophytes* ergosterol contents observed may be associated with quercetin, for which several derivatives were identified in ABT. In fact, quercetin has been shown to downregulate the fatty acid synthase reducing ergosterol levels, consequently causing plasma membrane disruption [62]. The fungal cell wall, mainly composed of chitin and glucan, is another fundamental fungal structure for fungal survival, playing a role in several crucial processes, amongst them being morphogenesis, cell function or adaptation to stress [63,64,65]. In response to the ABT, the chitin contents of *T. rubrum* cell wall, decreased relatively to the control but did not change significantly in *T. mentagrophytes* and *M. canis.* However, the β-1,3-glucan cell wall contents of the three dermatophytes decreased in response to ABT. This decrease was higher in *T. rubrum,* also the most sensitive to ABT, when compared with the other dermatophytes tested. These results suggest that the ABT extract targets the synthesis of β-1,3-glucan, supporting the possible mechanism of antifungal effect on dermatophytes. A decrease in β-1,3-glucan was also observed previously in *T. rubrum* when this fungus was treated with caffeine (our unpublished results). In fungi, β-1,3-glucan is synthesized by the β-1,3-glucan synthase. This enzyme is inhibited by a class of clinically available antifungals, the echinocandins, semi-synthetic cyclic lipopeptides, that comprises caspofungin, micafungin, anidulafungin and rezafungin [66,67]. They have the disadvantage of requiring intravenous administration because of the low absorption by the gastrointestinal tract, but they cause milder side effects compared to another systemic antifungal [66,67]. Echinocandins are fungicidal against many fungi including *Candida* spp. and fungistatic to the *Aspergillus* genus but are not clinically used to treat dermatophytosis. In filamentous fungi, a synergy between these compounds and drugs targeting chitin synthesis have been described [68,69]. Following these lines of evidence, the compound from ABT that inhibits β-1,3-glucan would be a pleasant addition to the antifungals used to treat dermatophytosis. Moreover, ABT showed an additive effect with terbinafine, meaning the effect of ABT targeting glucan synthesis adds to the effect of terbinafine, an inhibitor of ergosterol synthesis. 

The impact of ABT on the dermatophytes’ ultrastructure, observed by TEM, include an inner cell wall less electron-dense, probably associated with the decrease in β-1,3-glucan content. In relation to mitochondria, these are less abundant in *M. canis* exposed to ABT, while in *T. rubrum*, the mitochondrial morphology is disturbed. It is known that defects in mitochondria leads to an excess of reactive oxygen and a reduction in energy expenditure [70]. Moreover, mitochondria are linked to antifungal tolerance, as its dysfunction is associated with susceptibility to plasma-membrane-targeting drugs, which may also explain the additive effect that was observed with terbinafine [71]. In response to ABT, the vacuolization is also affected, and in *T. rubrum*, some vacuoles contain debris, while others are autophagy like-vacuoles. The increasing formation of cytoplasmic vacuoles may be a reflex of premature aging of the fungal cells, as pointed out by Müller and collaborators, 1992 [72]. 

The present work is the first report about the antidermatophytic action of Azorean Black Tea, and an approach to the mechanism of action. ABT is fungicidal against *T. mentagrophytes*, *T. rubrum* and *M. canis*, and has an additive effect with terbinafine. ABT impacts in the cell wall organization, becoming less electron-dense, as observed by TEM. ABT may also cause an impact in the energy conversion in the cell, affecting mitochondria viability. 

Considering the current limitations of the approved antifungal agents for the treatment of dermatophytosis, the development of innovative strategies and the search for new therapeutic targets, which combines safety with efficacy, is undoubtedly a current medical need [73]. The low cost and the expected low potential adverse effects, indicates that the formulation of black-tea-based phytotherapeutic products for topical application can be a complementary approach to the antifungals currently recommended. The black tea extract of *C. sinensis* contains components, such as catechin derivates, including epigallocatechin gallate, that have hydrophobic characteristics, which improve their retention within the skin when applied together with cream [74]. The development of products with tea extracts includes creams, lotions, shower gels and hair products [74]. Therefore, the present work is an opportunity for the development of topical formulations based in the combination of ABT and conventional antifungals clinically used for the treatment of dermatophytosis to improve the healing of these skin infections and decrease the toxicity associated with these drugs, as well as to open novel perspectives for the implementation of a clinical trial to test the topical use of ABT as a novel therapeutic approach to dermatophytosis. These results also introduce the scientific basis for the antifungal dermo-cosmetic potential of tea derivatives in general as a prophylactic strategy to avoid skin infection development, although further insights are needed to unravel the combined mechanisms of action of the several compounds found in black tea.

## 4. Materials and Methods

### 4.1. Crude Aqueous Extracts of Tea

The Azorean Black Tea (ABT) Pekoe was obtained from the Porto Formoso Factory (Pacheco e Mendonça, Lda., Estrada regional 9625-413, Porto Formoso, Azores). Crude aqueous infusions were made by adding 1 g of tea herbs to 100 mL boiling sterile distilled water, allowing the suspension to stand for 5 min under stirring with a magnet. Then, the solid tea herbs were removed by filtration through a sieve. The infusions obtained were either used immediately or frozen at −20 °C. The containers were wrapped in aluminum to avoid exposure to light, as the extract may be photosensitive. 

The dry weight of the ABT extracts were determined to obtain the mass corresponding to 1 mL of aqueous extract, by drying 1 mL of ABT extract at 50 °C overnight. The weighing was performed daily until obtaining a constant weight, to determine the concentration of ABT extract as well as the yield of the extraction. The extracts prepared were diluted to a working concentration of 4000 µg/mL.

### 4.2. Phytochemical Analysis

The analysis of the ABT extract was performed by high-performance liquid chromatography with a photodiode array detector coupled to mass spectrometry with Electrospray ionization (HPLC-PDA-ESI/MSn). This analysis was performed in a Surveyor liquid chromatograph equipped with a PDA detector and in interface with a Finnigan LCQ Advantage Ion Max mass spectrometer (Thermo Fisher Scientific, Waltham, MA, USA), equipped with an ESI ionization chamber. Chromatographic separation was performed at 20 °C on a C18 Spherisorb ODS-2 reverse-phase column (150 × 2.1 mm; particle size 3 μm; Waters Corp., Milford, MA, USA) preceded by a Spherisorb ODS- 2 C18 (10 × 4.6 mm; particle size 5 μm; Waters Corp., Milford, MA, USA). The mobile phase consisted of an aqueous solution of 2% formic acid (solvent A) and acetonitrile (solvent B). The gradient profile used was 0–40% (75 min) of solvent B at a flow rate of 200 μL/min. The first detection was made with a PDA detector using 280 and 320 nm as preferred wavelengths and the second detection was made in the mass spectrometer. Mass analysis was operated in the negative mode and positive ion mode and was programmed to perform a series of three scans: a full mass (MS) and a MS2 and MS3 scan of the most abundant ion. The collision gas was helium with a normalized collision energy of 35%. Nitrogen was used as nebulizing gas, with a sheath gas flow of 35 (arbitrary unit) and an auxiliary gas flow of 20 (arbitrary unit). The capillary temperature was set at 275 °C. The source voltage was set at 5 kV. The capillary voltage was set at 40 and −35 V, in positive and negative ion mode, respectively. Data treatment was performed with XCALIBUR software, version 4.0 (Thermo Fisher Scientific, Waltham, MA, USA).

### 4.3. Fungal Strains and Culture Conditions

Three clinical isolates of fungal species from our culture collection were tested: *T. rubrum* (IMF028), *T. mentagrophytes* (IMF029) and *M. canis* (IMF035). They were obtained from CYC-UC (Clinical Yeast Collection—University of Coimbra). *Trichophyton* spp. were cultured in potato dextrose agar (PDA, Difco), and *M. canis* was cultured on rice agar, at 30 °C. 

### 4.4. Antidermatophytic Activity of ABT Extract

The minimum inhibitory concentration (MIC) causing the growth inhibition of the selected dermatophytes was determined by microdilution following the E.DEF 9.3.1 EUCAST standards. The antifungals terbinafine (Acros Organics^TM^, Geel, Belgium) and griseofulvin (Sigma Aldrich, St. Louis, MO, USA), clinically used to treat infections caused by dermatophyte fungi, were used as the control. The MIC was measured as the lowest concentration for which the antifungals and ABT resulted in the inhibition of fungal growth.

The minimum fungicidal concentration (MFC) was tested from the culture at the MIC, 2xMIC and 4xMIC. In this procedure, 20 µL of the suspension of the wells, including from the growth control (extract-free medium) were inoculated in PDA and incubated for two weeks at 30 °C [75]. The MFC considered in this study was the lowest drug concentration for which no growth was observed.

### 4.5. Synergy Testing Assays

The impact of the combination of ABT with clinical antifungal agents, terbinafine and griseofulvin on antifungal efficiency was evaluated using the checkerboard assay method [76]. The quantification of this effect was performed by calculating the Fractional Inhibitory Concentration Index (FICI) value, expressed by the following equation: FIC Index = MIC_AB_/MIC_A_ + MIC_BA_/MIC_B_, where MIC_AB_ and MIC_BA_ are the MICs of each compound in combination, and MIC_A_ and MIC_B_ are the MICs of each drug individually. The impact on the drug potency was defined as synergistic when FICI ≤ 0.5, additive when 0.5 < FICI ≤ 1, indifferent when 1 < FICI < 2 and antagonist when FICI ≥ 2 (EUCAST Definitive Document E.Def 1.2) [77]. The plates were incubated at 35 °C, and the results were observed visually after 4 days.

### 4.6. Determination of the Modulation of Fungal ß-1,3-glucan, Chitin and Ergosterol in Response to ABT

The β-1,3-glucan, chitin and ergosterol contents were quantified in the three fungi under study. *T. rubrum, T. mentagrophytes* and *M. canis* were grown in two conditions: (1) YME liquid medium (0.4% of yeast extract, 1% of glucose and 1% of maltose extract) and (2) YME liquid medium with ABT extract at the MIC values. 

100 mL of YME liquid medium was inoculated with 100 µL of a spore suspension of 10^6^ spore/mL of each fungus and incubated at 30 °C in the dark at 120 rpm for 5 days. Then, the fungi were collected with a strainer, washed with H_2_O and squeezed with a spoon spatula to drain the water. Then, the fungi were freeze-dried, aliquoted in 15 mg aliquots and kept at −20 °C for the quantification assays of the different components.

The quantification of β-1,3-glucan levels in fungal cell walls were performed using the aniline blue assay, as described before [78]. Triplicate samples of 15 mg of each fungus grown in the ABT and control conditions, previously lyophilized, were used. The mycelium was sonicated in 1 M NaOH, and the β-1,3-glucan concentration was determined by aniline blue fluorescence at 405 nm excitation and 460 nm emission wavelengths in a fluorimeter (Spectra Max^®^ ID3, Molecular Devices, San Jose, CA, USA) [78,79]. 

The quantification of the cell wall chitin was performed by measuring the glucosamine released by acid hydrolysis of purified cell walls from the previously prepared 15 mg aliquots, as described before [79,80]. The absorbance was read at 520 nm on a plate reader using a SpectraMax^®^ Plus 384 spectrophotometer (Molecular Devices, San Jose, CA, USA).

The ergosterol extraction and quantification was carried out in 15 mg of the fungal samples previously prepared with the alcoholic KOH method following the protocol described previously [81] with minor modifications [82]. The spectral absorbance range between 220 and 300 nm was obtained in a plate reader using a SpectraMax^®^ Plus 384 spectrophotometer.

### 4.7. Characterization of Morphological and Ultrastructural Changes

The ultrastructural changes induced by the ABT extracts in *T. rubrum, T. mentagrophytes* and *M. canis* were analyzed by Transmission Electron Microscopy (TEM). To perform TEM analysis, the inoculum was prepared as described in Section 2.4, except that the mycelia of the fungi were not lyophilized. The samples were fixed with 2.5% glutaraldehyde in 0.1 M sodium cacodylate buffer (pH 7.2). Postfixation was performed using 1% osmium tetroxide for 1 h. After rinsing with the buffer, the samples were dehydrated in a graded ethanol series (30 to 100%), impregnated and embedded in epoxy resin (Fluka Analytical^®^, Rahway, NJ, USA). Ultrathin sections (80 nm) were mounted on copper grids (300 mesh) and stained with uranyl acetate 2% (15 min) and 0.2% lead citrate (10 min). TEM images were obtained using a FEI-Tecnai^®^ G2 Spirit Bio Twin™ transmission electron microscope (FEI, Hillsboro, OR, USA).

### 4.8. Statistical Analysis 

The analysis of the data was performed in GraphPad Prism (version 8.0.1.244) software, and the results were presented as means ± SEM, using multiple *t*-tests for multiple comparison tests to see the statistical differences, with *p* < 0.05 being considered statistically significant. 

## Figures and Tables

**Table 1:** Compounds identified in ABT extract by HPLC-PDA-ESI/MSn.

Peak	Identification	R_t_ (min.)	λ_max._ by HPLC/PDA (nm)	^a^ [M − H]/ ^b^ [M − H]^+^	MS^2^	MS^3^
1	Isocitric acid	2.14	261	^a^ 191 (100)	[191]: 191 (75),173 (45), 155 (15), 111 (100)	[191 111]: 111 (100)
2	Galloylquinic acid	3.06	271	^a^ 343(100)	[191]: 191 (100), 169 (25)	-
3	Gallic acid	3.20	270	^a^ 169 (100)	[169]: 169 (70), 125 (100)	[169 125]: 125 (100)
4	Theobromine	5.29	273	^b^ 181(100)		-
5	galloyl-(epi)gallocatechin-(epi)gallocatechin	5.35	273	^a^ 761 (100)	[761]: 609 (40), 591 (100)	[761 591]: 591 (50), 573 (100)
6	(Epi)gallocatechin	6.27	278	^a^ 305 (100)	[305]: 261 (17), 221 (45), 219 (55), 179 (100)	-
7	Galloyl-HHDP-glucose	7.55	276	^a^ 633 (100)	[633]: 633 (50), 463 (10), 301 (100)	[633 301]: 301(100)
8	4-p-coumaroylquinic acid	11.16	311, 296	^a^ 337 (100)	[337]: 173 (100)	[337 173]: 173 (85), 111 (100), 93 (83)
9	Epigallocatechin gallate	12.17	280	^a^ 457 (100)	[457]: 331 (60), 305 (40), 287 (20), 169 (100)	[457 169]: 169 (100), 125 (70)
10	Gallocatechin gallate	12.81	279	^a^ 457 (100)	[457]: 331 (60), 305 (40), 287 (20), 169 (100)	[457 169]: 169 (100), 125 (50)
11	Caffein	13.38	273	^b^ 195 (100)	-	-
12	4″-*O*-Glucosylvitexin	13.95	272	^a^ 593 (100)	[593]: 593 (30), 575 (20), 503 (20), 473 (100)	[593 473]: 383 (20), 353 (100)
13	6-pentosyl–8 hexosyl–apigenin	18.43	273, 342	^a^ 563 (100)	[563]: 563 (60), 545 (40), 503 (80), 473 (100), 383 (35), 353 (40)	[563 473]: 383 (20), 353 (100)
14	6-pentosyl–8 hexosyl–apigenin	19.03	273, 342	^a^ 563 (100)	[563]: 563 (45), 545 (40), 503 (55), 473 (100), 443 (75), 383 (30), 353 (25)	[563 473]: 383 (15), 353 (100)
15	6-pentosyl–8 hexosyl–apigenin	19.68	273, 342	^a^ 563 (100)	[563]: 563 (50), 545 (20), 503 (25), 473 (50), 443 (75), 383 (100), 353 (20)	-
16	Epicatechin gallate	20.04	275	^a^ 441 (100)	[441]: 289 (100), 169 (20)	[441 289]: 289 (40), 245 (100), 205 (25), 179 (20)
17	Catechin gallate	20.85	274	^a^ 441 (100)	[441]: 289 (100), 169 (20)	[441 289]: 289 (15), 245 (100), 231 (15), 205 (25), 179 (15)
18	Quercetin-3-*O*-galactosyl-rhamnosyl-glucoside	22.00	253sh, 266m 290sh, 350	^a^ 771 (100)	[771]: 771 (90), 609 (40), 301 (100)	-
19	Quercetin-3-*O*-galactosyl-rhamnosyl-glucoside	23.01	253sh, 266m 290sh, 350	^a^ 771 (100)	[771]: 771 (100), 301 (40)	-
20	Quercetin-3-*O*-galactosyl-rhamnosyl-glucoside	23.56	257sh, 264m 291sh, 352	^a^ 771 (100)	[771]: 771 (70), 301 (40)	-
21	Quercetin-3-*O*-rutinoside	24.72	257sh, 264m 291sh, 352	^a^ 609 (100)	[609]: 609 (100), 301 (100)	-
22	Quercetin-3-*O*-rutinoside	25.30	257sh, 264m 291sh, 352	^a^ 609 (100)	[609]: 609 (100), 301 (100)	-
23	Kaempferol-3-*O*-glucosyl-rhamnosyl-glucoside	26.68	265, 299sh, 347	^a^ 755 (100)	[755]: 755 (40), 593 (15), 285 (100)	-
24	Kaempferol-3-*O*-glucosyl-rhamnosyl-glucoside	27.13	265, 299sh, 347	^a^ 755 (100)	[755]: 755 (40), 593 (15), 285 (100)	-
25	Kaempferol-3-*O*-rutinoside	28.67	265, 299sh, 347	^a^ 593 (100)	[593]: 593 (20), 285 (100)	-
26	Kaempferol-3-*O*-rutinoside	29.32	265, 299sh, 347	^a^ 593 (100)	[593]: 593 (20), 285 (100)	-
27	Kaempferol-3-*O*-galactoside	29.79	265, 299sh, 347	^a^ 447 (100)	[477]: 447 (100), 285 (60)	-
28	Kaempferol-3-*O*-glucoside	30.33	265, 299sh, 347	^a^ 447 (100)	[477]: 447 (100), 285 (60)	-
29	Quercetin-3-(2G-*p-coum-trans*-3G)-2G-arabinosyl-3R-rhamnosyl rutinoside	41.82	253sh, 269, 290sh, 359	^a^ 1033 (100)	[1033]: 887 (100)	[887 741]: 741 (20), 301 (100)
30	Theaflavin gallate	44.67	270, 359	^a^ 715 (100)	[715]: 577 (15), 563 (50), 545 (75), 527 (100), 501 (30), 419 (30)	-
31	Theaflavin gallate	45.98	270, 356	^a^ 715 (100)	[715]: 697 (40), 563 (100), 545 (75), 527 (55), 483 (55), 407 (45)	-

**Table 2:** MIC, MFC and FICI values of with *Trichophyton* spp. and *M. canis* with Azorean Black Tea, terbinafine and griseofulvin.

	ABT		Terbinafine			Griseofulvin		
Fungal Species	MIC	MFC	MIC	FICI *	Index	MIC	FICI **	Index
*T. mentagrophytes*	250.0	250.0	0.0312	0.75	Additive	0.5	1.5	Indifference
*T. rubrum*	125.0	125.0	0.0625	1	Additive	0.5	1.5	Indifference
*M. canis*	500.0	500.0	0.0312	0.75	Additive	0.5	1.5	Indifference

* FICI—Combination of terbinafine and ABT; ** FIC—combination of griseofulvin and ABT.

## Data Availability

Data are contained within the article and supplementary materials.

## Supplementary Materials

The following supporting information can be downloaded at: https://www.mdpi.com/article/10.3390/molecules28237775/s1, Figure S1: Total ion current chromatogram of ABT. Peaks 1–31 are listed in Table 1.
