# Supplementary figures and images for: Azorean Black Tea (Camellia sinensis) Antidermatophytic and Fungicidal Properties
Source: Molecules. 2023 Nov 25;28(23):7775. doi: 10.3390/molecules28237775 (PMC10707949; doi:10.3390/molecules28237775)

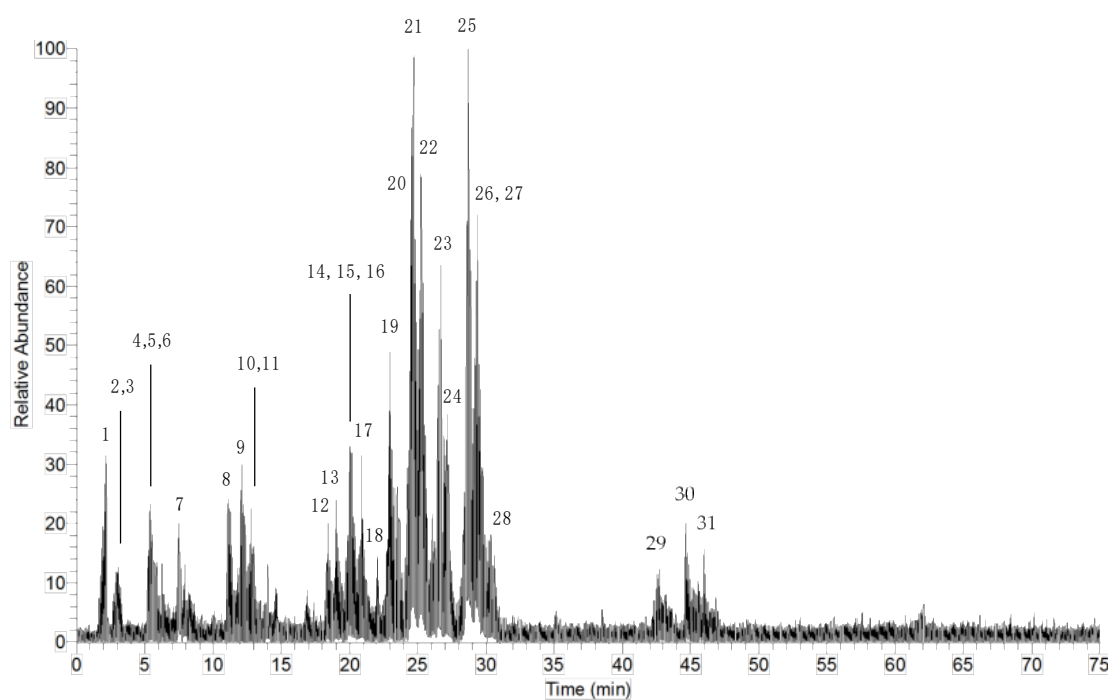

**Figure S1.** Total ion current chromatogram of ABT. Peaks 1–31 are listed in Table 1.

Supplement: Supplementary file 1 [file molecules-28-07775-s001.zip › molecules-2709182-supplementary.pdf]
